# *Arquivos de Neuro-Psiquiatria* , 80 years: part 2 (1963–1982)

**DOI:** 10.1055/s-0045-1815737

**Published:** 2026-03-11

**Authors:** Rodrigo Fagundes da Rosa, Francisco Duque Paiva Giudice Junior, Alan Duarte Braz, Eduarda Domingo da Silva Lacerda, Nayana Freire de Almeida Fontes, Anny Kariny Rodrigues dos Santos, Francisco de Assis Ferreira Júnior, João Victor Uchôa Sales, Francisco de Assis Aquino Gondim

**Affiliations:** 1 Universidade Federal do Ceará, Faculdade de Medicina, Fortaleza CE, Brazil.; 2 Hospital Geral de Fortaleza, Fortaleza CE, Brazil.; 3 Universidade Federal do Ceará, Faculdade de Medicina, Departamento de Medicina Clínica, Fortaleza CE, Brazil.

**Keywords:** Arquivos de Neuro-Psiquiatria, Neurology, History, Periodical

## Abstract

**Background:**

*Arquivos de Neuro-Psiquiatria* (ANP) celebrated 80 years in 2023. We have previously evaluated the publication trends throughout the first 20 years.

**Objective:**

To analyze the publication trends, authorship, and editorial patterns of the volumes 21 to 40 of ANP (1983–1962).

**Methods:**

Data were tabulated independently by two blinded researchers and cross-verified by independent researchers and automated data extraction technology (Python script).

**Results:**

From 1963 to 1982, 20 volumes, 80 issues, and 904 articles were published. In 1970, ANP became the official journal of Academia Brasileira de Neurologia (ABN, the Brazilian Academy of Neurology). We analyzed 795 articles (and excluded non-research papers). Compared to the first 20 years, there was a significant increase in the total number of authors per article (2.73; *p* < 0.05), a significant increase in the percentage of female authors (to 10.96%; *p* < 0.05), and a decrease in the number of pages per article ( *p* < 0.05). Antonio Spina-França, Walter Carlos Pereira, and Michel Pierre Lison were the most prolific authors. Most of the articles were on the topics of Neurology/Child Neurology, with a decrease in the percentage of Psychiatry papers and an increase in Basic Research contributions. There was also a linear increase in the total number of articles from 1963 to 1982, as detected by regression analysis (R ^2^ = 0.86; *p* < 0.0001). Most of the articles were written in Portuguese and by authors from Southeastern Brazil (71.1%).

**Conclusion:**

The years 1963 to 1982 marked the resilience of ANP during the tempestuous years of the Brazilian Military dictatorship, coinciding with an increased split between the Neurology and Psychiatry fields, the appearance of clinical trials and large case series, and a greater development in the understanding of neuroinfectious diseases.

## INTRODUCTION

*Arquivos de Neuro-Psiquiatria* (ANP) was founded in 1943; 1 since then, it has been the leading Brazilian medical journal in the field of Neurology. We have previously described the characteristics of its first 20 years (1943–1962) as part of a 4-part project with the aim of evaluating its history throughout the first 80 volumes. 1

In the first 20 volumes, professor Osvaldo Lange (from Universidade de São Paulo – USP) served as Chief Editor. 1 2 Most of the articles were written in Portuguese and were by authors from Southeastern Brazil, although there were international contributions from Egaz Moniz, Barraquer-Bordas, Bing, Denny-Brwon, and Wartenberg. 1 In 1962, Academia Brasileira de Neurologia (ABN, the Brazilian Academy of Neurology) was founded and greatly influenced the destiny of ANP. However, only in 1970 ANP became ABN's official journal. 2

The advances in neuroscience led to a progressive split between Neurology and Psychiatry as distinct fields. Within ANP itself, there was a proportional decrease in Psychiatry articles throughout its first 20 years. 1 The aim of the present study is to continue the investigation of the ANP publication trends from 1963 to 1982, also taking into account the major historical and scientific developments of this period.

## METHODS

We analyzed all 20 volumes published by ANP from 1963 to 1982. Each article from the internet-based repository was individually reviewed, as well as all physical editions available at the Professor Jurandir Marães Picanço Health Sciences Library, at Universidade Federal do Ceará, as previously described. 1 Data from both sources were tabulated by five independent and blinded researchers. Subsequently, two researchers (RFR and FDPGJ) cross-verified the collected data. In this manuscript, after the cross-verified data collection, we also incorporated the use of an automated data extraction technology as a second way to cross-verify the extracted data. Therefore, we used a Python script developed by one of the researchers (ADB)—improved with the aid of teo other researchers (RFR and FDPGJ)—to specifically capture the information directly from the journal's website pages, according to the structure and classifications provided by the publication itself. Following the automated extraction, the authors manually reviewed the data and cross-verified it against data they had previously extracted manually.

The following parameters were analyzed in each article: year of publication, number of authors, number of pages, city/state/country of the institution that submitted the article, gender of the first author, and article type (original article, case report, practical note, conference, technical note). 1 Articles from the following categories were excluded from the analysis (except to express general statistical figures), since they frequently did not list the author/institution: book reviews, magazine reviews, errata, homages, scientific meetings, and obituaries (in memoriam). 1 We also evaluated the total number of citations from each article as detected by Dimensions AI (Digital Science & Research Solutions Ltd), available at the ANP website. 1 Descriptive statistics was conducted to detail the most important aspects of the publication (expressed as mean+standard deviation values), and the Chi-squared and two-tailed *t* -test were used to compare the trends in editorial findings throughout the first 40 ANP volumes. 1

## RESULTS

Between 1963 and 1982, 80 issues were published in 20 volumes of the journal. A total of 904 articles (of all types) were published. During this period, four issues were published annually. Out of the 904 articles, 795 were original scientific articles (including research communications and clinical case reports). The remaining items (excluded articles) consisted of 76 book reviews, 3 magazine reviews, 15 in-memoriam articles (obituaries or tributes), and 15 errata.

**Figure 1.**
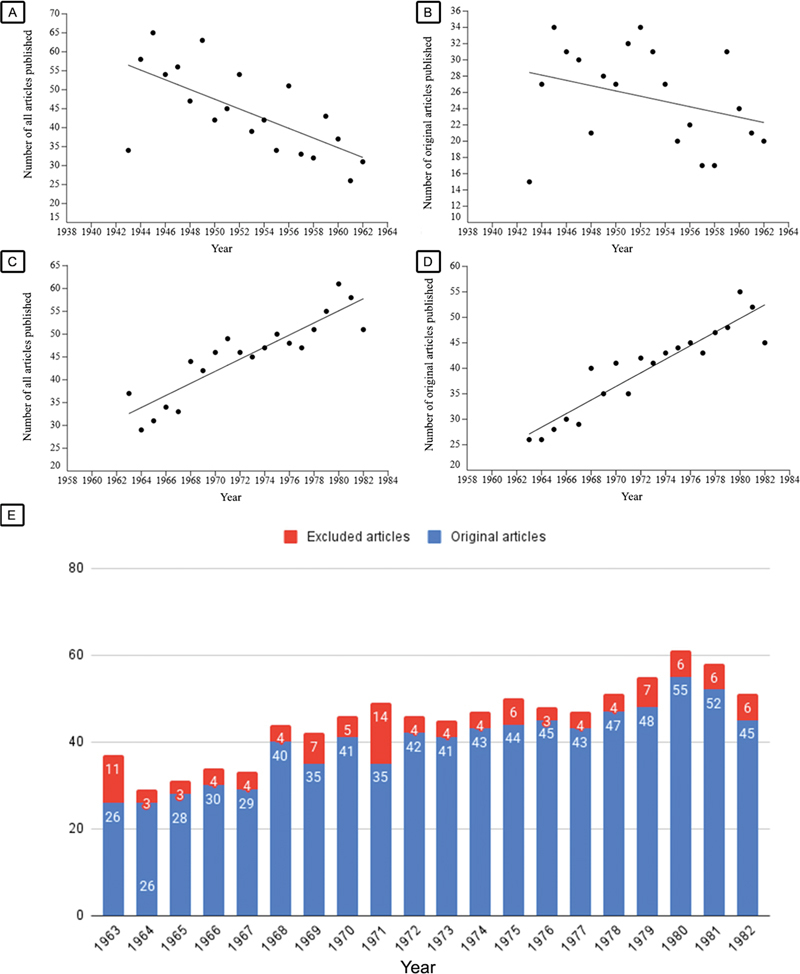
( **A** ) Linear regression analysis of the total number of articles published between 1943 and 1962 (first 20 volumes of *Arquivos de Neuro-Psiquiatria* , ANP). ( **B** ) Linear regression analysis of the total number of articles analyzed from 1943 to 1962. ( **C** ) Linear regression analysis of the total number of articles published between 1963 and 1982 (the second 20 volumes of ANP). ( **D** ) Linear regression analysis of the total number of articles analyzed per year from 1963 to 1982. ( **E** ) Number of analyzed (blue) and non-analyzed (red) articles published per year from 1963 to 1982.

After excluding 109 articles, a total of 795 papers were analyzed (636 “original articles”, 155 “case reports”, 2 “conferences”, 1 “practical note”, 1 “technical note”). We identified 2,171 authors, comprising 2.73 authors per article. There was a statistically significant increase in the number of authors per article in comparison with the first 20 volumes (2.73 versus 1.78 respectively; *p* < 0.00001). The mean numbers of all article subtypes and all original articles published per year during these 2 decades were of 45.2 + 8.7 and 39.8 + 8.5, respectively. There was a significant increase in the number of original articles published from 1963 to 1972 versus from 1973 to 1982: 33.2 + 6.2 versus 46.3 + 4.4, respectively ( *p* = 0.000018). Figure 1 details the trends regarding the total number of publications and original articles in the first 40 years of ANP by regression analysis. Figure 1E details the total number of publications (of all articles subtypes) and of original articles by each year from 1963 to 1982. As Figures 1C,D show, there was a significant increase in total number of publications (of all articles subtypes) and of original articles from 1963 to 1982, in which y corresponds to the total number of publications or of original articles, and x, to the year: y = -2567.9917 + 1.3248x (R ^2^ = 0.81; *p* < 0.001) and y = -2592.7218 + 1.3346x (R ^2^ = 0.86; *p* < 0.001) respectively. This contrasts with the significant decrease in publications (of all article subtypes) and of original articles detailed by regression analysis throughout the first 20 years (1943–1962; Figures 1A and 1B ): y = 2532.5865 - 1.2744x (R ^2^ = 0.45; *p* < 0.01, from 1943–1962) and y = 1122.4211 - 0.5614x (R ^2^ = 0.32; *p* < 0.01, from 1944–1962).

We observed a decrease in the mean number of pages per article among the original articles analyzed during the period, from 7 ± 4.2 (range: 2–38) pages in comparison with the period from 1943 to 1962 (11.9 ± 6.7 pages; *p* < 0.00001). The mean number of pages in volumes 21 to 30 was similar in comparison with volumes 31 to 40: 7 ± 5.2 versus 7 ± 3.6 pages ( *p* = 0.33). From 1963 to 1982, the mean number of citations per article was of 2.18 ± 4.2 (similar to the mean of 2.64 ± 5.55 citations observed from 1943–1962). There was no significant increase in the number of citations per article when comparing the first 10 with the last 10 volumes: 2.11 ± 4.8 versus 2.24 ± 3.5 respectively ( *p* = 0.5668).

Professor Antonio Spina-França stands out as the most prolific author, followed by Walter Carlos Pereira and Michel Pierre Lison. Table 1 further details the top 10 most productive authors in volumes 21 to 40. We also observed an increase in the percentage of female contributions (as authors/coauthors) in comparison with the first 20 volumes (total of 137 female authors): 238 (10.96%) versus 3,75% ( *p* < 0.05) respectively. The lower part of Table 1 also lists the most prolific female authors in this period, which was led by Lígia M. Barbosa Coutinho, Valeriana Moura Ribeiro, and Eneida Baptistete Matarazzo. According to the fields of study, there were 507 Neurology articles, 127 Child Neurology papers, 119 Neurosurgery articles, 36 Psychiatry articles, and 36 Basic Research Papers. Table 2 further details the fields of study of the subspecialties of the Neurology, Neurosurgery and Child Neurology articles (each article could have more than one subspecialty listed). During the period analyzed, 99 case series and 37 drug trials were identified. Table 3 also details the 10 articles with the highest number of citations between 1963 and 1982.

**Table 1:** List of the 10 most prolific authors from volumes 21 to 40 of *Arquivos de Neuro-Psiquiatri* *a* (ANP, 1963–1982) and of the most prolific female authors

N°	Author	Authorships	Coauthorships	Total*
1	Antonio Spina-França	26	27	53
2	Walter Carlos Pereira	26	10	36
3	Michel Pierre Lison	13	18	31
4	Gilberto Machado de Almeida	17	12	29
5	Luís Marques de Assis	15	6	21
6	Horácio M. Canelas	11	10	21
7	Antonio Branco Lefèvre	4	17	21
8	Wilson Luiz Sanvito	16	3	19
9	Lineu Cesar Werneck	16	3	19
10	José Lamartine de Assis	10	8	18
	**Female authors**			
1	Lígia M. Barbosa Coutinho	7	5	12
2	Valeriana Moura Ribeiro	5	4	9
3	Eneida Baptistete Matarazzo	4	2	6
4	Olga P. Sanz	2	3	5
5	Eliana C. F. O. Wanderley	1	4	5
6	Gilda Kasting	0	5	5
7	Rosa Helena Longo	1	4	5
8	Maria Irmina Valente	1	4	5
9	Anamarli Nucci	0	4	4

Note: *Main authorships plus coauthorships.

**Table 2:** List of the main neurological topics covered in the articles analyzed from 1963 to 1982

Main topics	Number of articles
Neuroinfection	111
Neuromuscular	83
Epilepsy	80
Neuro-oncology	77
Neurogenetics	66
Neurovascular	56
Cerebrospinal fluid	54
Clinical neurophysiology	49
Movement disorders	29
Others	25
Neuroimaging	24
Neuroimmunology	24
Myelopathy	21
Peripheral neuropathy	21
Behavioral neurology/dementia	16
Inborn errors of metabolism	16
Traumatic brain injury	6
Myopathy	6
Headache	5
Neuromuscular junction	5
Neurocritical care	4
Sleep medicine	4

**Table 3:** List of the 10 most cited articles in volumes published between 1963 and 1982, with the total number of citations

N°	Article	Citations*
1	França et al. (1969) | Chronic Chagas disease associated with lymphatic leukemia: occurrence of acute encephalitis as a possible alteration of the immune state 14	36
2	Pereira et al. (1965) | [Neurological lesions in South American blastomycosis. Anatomopathological study of 14 cases] 15	35
3	Raphael (1966) | Localização nervosa da blastomicose sul-americana 16	31
4	Canelas et al. (1963) | Cysticercosis of the nervous system: less frequent clinical forms. III. Spinal cord forms 17	30
5	Wittig et al. (1968) | Neuroblastomycosis: report of three cases 18	25
6	Pereira et al. (1977) | [Bifrontal decompressive craniotomy as the treatment for severe cerebral edema] 19	24
7	Pereira et al. (1965) | [Brain localization of South American blastomycosis. Considerations apropos of 9 cases] 20	23
8	Reimão and Lefèvre (1982) | Evaluation of flurazepam and placebo on sleep disorders in childhood 21	23
9	Scaff et al. (1971) | [Schistosomal meningoradiculomyelopathy] 22	22
10	Spina-França et al. (1980) | [Myelopathies: diagnostic aspects] 23	22

Note: *Detected by Dimensions AI (Digital Science & Research Solutions Ltd), available at the website of *Arquivos de Neuro-Psiquiatria* (ANP).

**Figure 2.**
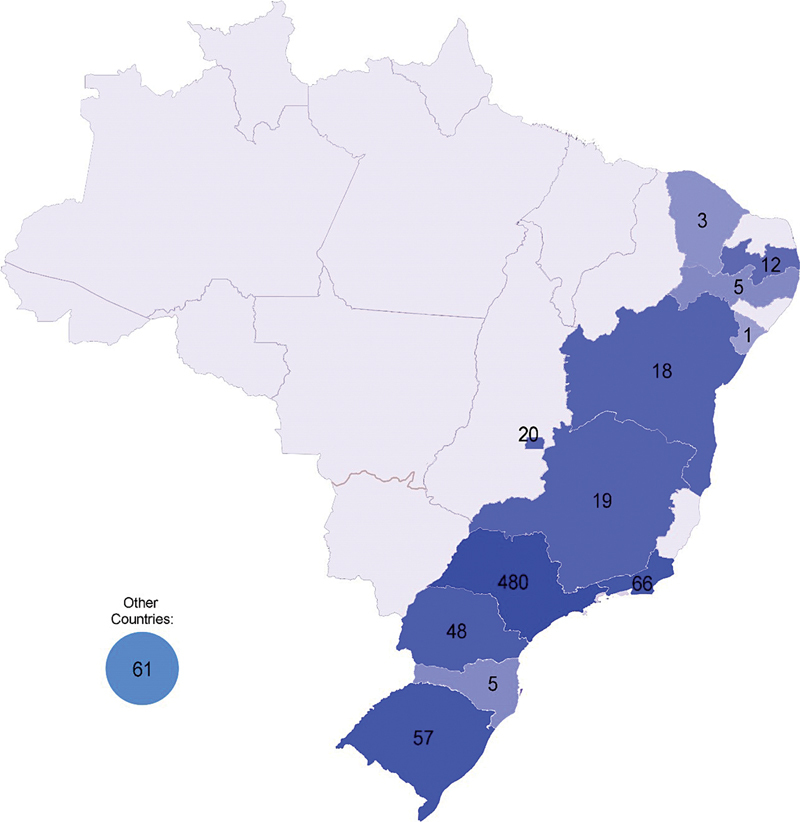
Map of Brazil in 1982, with the number of articles from each Brazilian state and the total number of articles written by foreign authors (international articles).

Figure 2 details the geographical distribution of publications from Brazil: most articles were form Southeastern Brazil (71.1%). Only 4.9% were from the Northeastern, 2.5% from the Midwestern, and 13.8% from the Southern region of Brazil. No original articles were from Northern Brazil, and 61 (7.6%) foreign articles came from the following countries: Germany (15), Argentina (12), Spain (9), the United States of America (9), England (3), Peru (3), Canada (3), Chile (2), France (1), Denmark (1), Austria (1), Israel (1), and Venezuela (1). Most articles were published in Portuguese (87.3%). Few articles were published in English (9.2%) and Spanish (3.5%).

## DISCUSSION

As we have previously discussed, the first 20 volumes of ANP (1943–1962) established it as a vital platform to disseminate neuroscientific knowledge within the Brazilian neurological community, quickly surpassing the importance of earlier publications: *Arquivos Brasileiros de Psiquiatria, Neurologia e Ciências Afins* and *Revista Neurobiologia* . 1 Six years after the foundation of ANP in São Paulo, *Jornal Brasileiro de Neurologia* was founded as a scientific publication of Instituto de Neurologia Deolindo Couto (Neurology Institute of Universidade Federal do Rio de Janeiro) and Associação de Neurologia do Estado do Rio de Janeiro (ANERJ, the Neurological Association of the State of Rio de Janeiro), renamed *Revista Brasileira de Neurologia* in 1983.

On May 5th, 1962, ABN was founded at Instituto de Neurologia da Universidade do Brasil (currently called Instituto Deolindo Couto), in Rio de Janeiro. 3 The foundation meeting was chaired by Professor Deolindo Couto, who was acclaimed as patron of ABN during his lifetime. 3 The first ABN scientific meeting was held in the city of Curitiba, state of Paraná, in 1963. 3 Professor Deolindo Couto was also the first ABN president, and only in 1970 ANP became the official journal of ABN. 2

The year 1951 marks the foundation of Conselho Nacional de Pesquisas (CNPq, renamed in 1974 as Conselho Nacional de Desenvolvimento Científico e Tecnológico) and Coordenação de Aperfeiçoamento de Pessoal de Nível Superior (CAPES). Together with Financiadora de Estudos e Projetos (FINEP), founded in 1967, and Fundação de Amparo à Pesquisa do Estado de São Paulo (FAPESP), founded in 1962, those 4 institutions governed most of the Federal and State budgets devoted to the scientific enterprise in Brazil.

From 1943 to 1982 (the first 4 decades of ANP), the research articles published were mostly the result of the description of clinically-oriented observations (case reports and series), and they were not financially supported by the 4 most important Brazilian scientific agencies. This contrasts with other publications which initially were mostly devoted to basic science (such as the *Brazilian Journal of Medical and Biological Research* , founded in 1968 as *Revista Brasileira de Pesquisas Médicas e Biológicas* ).

The findings of the current study highlight the persistent publication trends observed during the first 20 years of foundation of ANP. 1 From 1963 to 1982, there was a decrease in the proportion of foreign publications and a slightly-greater contribution of the Northeastern and Southern Brazilian states, although most of the publications were still from Southeastern of Brazil, mainly from the state of São Paulo, the journal's birthplace and the operating location of its founders. The contribution of female authors was still limited (of approximately 11%), but it increased 4 times in comparison with the first 20 years. 1 Most of the papers were published in Portuguese (87.3%), reflecting the needs of the Brazilian neurologists to publish their own accounts in scientific journals, circumventing the language barriers from international publications. Overall, those trends reflect the scientific and medical challenges during the 21 years of Military dictatorship in Brazil (1964–1985). Despite those challenges, there was a continuous increase in the number of published papers over the 20-year period (documented by linear regression analysis; Figure 2 ) and a change in the paper format to modern standards: a decrease in the number of pages per article and an increase in the number of authors per article. Those last format trends can be similarly observed in the last decades of the twentieth century in most medical journals.

Table 3 (and also the number of papers within the different neurological topics in Table 2 ) shows that the most cited articles were related to neuroinfectious diseases (Chagas disease, neurocysticercosis, schistosomiasis, and fungal infections). This is also reinforced by the fact that Professor Antonio Spina-França stands out as the most prolific author, and his main expertise was cerebrospinal fluid (CSF) analysis. During the period analyzed, we could document the publication of 97 case series and 37 drug trials. In 2025, Barbosa et al. 4 detailed the contributions of Professor Horácio Canelas on the evaluation of patients with Wilson's disease, starting in the 1960s, when penicillamine was already in use in Europe. Their studies raised the status of USP as an international reference center for the study of Wilsońs disease, a medical illness that was first reported in Brazil by professor Austragésilo Filho in 1944, also in ANP. 5 The first study describing the use of head computed tomography in ANP appeared in December 1980. 6 To avoid the risk of selection bias about specific topics, imprecisions about the first disease descriptions in Brazil, especially considering that not all accounts of case reports are listed in PubMed or have digital object identifiers, we will not further elaborate on the merit of the publications during the period studied (1963–1982). Following the trends from 1953 to 1962, we did observe a decrease in the proportion of Psychiatry publications and a slight rise in Basic Science contributions. Those contributions included a review paper by neurophysiologist Miguel Rolando Covian 7 (originally from Argentina and from the same institute as Bernardo Houssay) from Faculdade de Medicina de Ribeirão Preto.

This period also marked the emergence of clinical drug trials and large case series in ANP. A significant number of the initial trials originated from Psychiatry, and they were conducted in the inpatient setting of sanatoriums, such as the 1967 trial on the antipsychotic thiothixene 8 and the 1969 trial on the antidepressant doxepin, 9 both from Sanatório Anhembi, in São Paulo. However, this trend extended to large-scale neurological studies from university hospitals. A notable example is a 1969 report by Luís Marques-Assis, 10 from Hospital das Clínicas da Faculdade de Medicina da Universidade de São Paulo (HCFMUSP), which analyzed the drug treatment outcomes in a cohort of 1,217 patients with epilepsy. The appearance of psychopharmacological trials and large clinical series signaled a new, more complex direction for the research published in ANP.

Lastly, the first publication about the standards of Neurological training comparing the curriculum from Brazilian and international medical schools (from South and North Americas, Europe, Asia, and Australia) was published in 1965. 11 This paper was presented at the First Brazilian Neurological Congress, in the city of Ribeirão Preto, state of São Paulo, which was held from July 26th to 31 ^st^ , 1964, by Professors Paulo Pinto Pupo and Jorge Armbrust-Figueiredo. This study 11 revealed that the Neuroscience/Neurological graduate training was more organized and homogeneous in the United States than in Europe, with major improvements after the Second World War. The same was true for Postgraduate Neurological training, which was still in its infancy in Brazil. 11 This paper served as an important document to establish the basis for formal neurological training sponsored by the Brazilian Ministry of Education. Also on the subject of Neurological education and Semiology, it is important to detail the paper written by Professor Barraquer-Bordas about the controversial concept of upper-extremity Babinski sign, 12 and a paper coauthored by Professor Spina-França comparing the traditional way of eliciting the Babinski sign with a method using cold temperature. 13

In summary, during the tempestuous years from 1963 to 1982, ANP remained resilient and continued to focus on its mission of enabling Brazilian neurologists to express their scientific ideas in their native language (Portuguese) and continued its split from Psychiatry and the trend to its modern format: higher number of authors per article, decrease in the length of the published papers, appearance of clinical trials and large case series, and greater development in the understanding of neuroinfectious diseases ( Table 3 ).

## References

[1] Rosa RFd Giudice FDdP Junior Gondim FdAA Arquivos de Neuro-Psiquiatria, 80 years: part 1 (1943-1962) Arq Neuropsiquiatr 2025 83 03 1 8 10.1055/s-0045-1804917 PMC11922621 40107258

[2] Teive H AG Caramelli P Arquivos de Neuro-Psiquiatria: 75 years Arq Neuropsiquiatr 2018 76 01 50 52 10.1590/0004-282x20170187 29364394

[3] Gomes MdM Cavalcanti JLdS Fifty years of the Brazilian Academy of Neurology Arq Neuropsiquiatr 2012 70 12 956 959 10.1590/s0004-282x2012001200011 23295426

[4] Barbosa E R Parmera J B Cury R G Cançado E LR Bonilha PÁAM Teive H AG Sixty years of the first studies by Horácio Martins Canelas on Wilson's disease Arq Neuropsiquiatr 2025 83 02 1 3 10.1055/s-0045-1802553 PMC11850080 39993444

[5] Austregésilo AFilho.Degeneração mucoide da oligodendroglia em um caso de enfermidade do tipo Wilson Arq Neuropsiquiatr 1944 2 02 170 176 10.1590/S0004-282x1944000200003

[6] Shibata M K Bianco E Moreira F A de Almeida G M. [Tumoral form of cerebral cysticercosis: diagnosis by computerized tomography.]Arq Neuropsiquiatr 1980 38 04 399 403 10.1590/s0004-282x1980000400010 7469830

[7] Covian M R [Physiopathogenesis of pain.] Arq Neuropsiquiatr 1965 23 03 143 164 10.1590/s0004-282x1965000300001 5856791

[8] Navarro E S Fonseca C A Baptistete E Penna J MR Saldanha M V Santos CCd Efeitos do tiotixene (P-4657 B) em crianças e adolescentes psicóticos Arq Neuro-Psiquiatr 1968 26 04 363 369 10.1590/s0004-282x1968000400008

[9] Marques-Assis L Martins C Ensaio clínico com novo antidepressivo (Doxepin) em pacientes internados Arq Neuropsiquiatr 1969 27 02 119 124 10.1590/s0004-282x1969000200006

[10] Marques-Assis L Considerações a propósito do tratamento medicamentoso de 1217 pacientes epilépticos: I: estudo em relação ao tipo de epilepsia e ao eletrencefalograma Arq Neuropsiquiatr 1969 27 04 312 320 10.1590/s0004-282x1969000400008

[11] Pupo P P Armbrustfigueiredo J [Training in neurology.] Arq Neuropsiquiatr 1965 23 03 205 219 10.1590/s0004-282x1965000300008 5856797

[12] Barraquer-Bordas L [Symptomatology of the pyramidal deficit at the level of the hand. A methodological note.] Arq Neuropsiquiatr 1975 33 01 61 63 10.1590/s0004-282x1975000100008 1164209

[13] Silva J AG Spina-França A Evaluation of cold stimulation tecnique for eliciting Babinski's toe Phenomenon Arq Neuropsiquiatr 1966 24 03 180 184 10.1590/S0004-282x1966000300004

[14] França L CM Lemos S Fleury R N Melaragno Filho R Ramos H A Jr Pasternak J Chronic Chagas disease associated with lymphatic leukemia: occurrence of acute encephalitis as a possible alteration of the immune state Arq Neuro-Psiquiatr 1969 27 01 59 66 10.1590/S0004-282x1969000100005

[15] Pereira W C Raphael A Sallum J [Neurological lesions in South American blastomycosis. Anatomopathological study of 14 cases.] Arq Neuropsiquiatr 1965 23 02 95 112 10.1590/s0004-282x1965000200005 5851897

[16] Raphael A Localização nervosa da blastomicose sul-americana Arq Neuro-Psiquiatr 1966 24 02 69 90 10.1590/s0004-282x1966000200001

[17] Canelas H M Ricciardi-Cruz O Escalante A D Cysticercosis of the nervous system: less frequent clinical forms. III. Spinal cord forms Arq Neuropsiquiatr 1963 21 77 86 10.1590/s0004-282x1963000200001 14076114

[18] Wittig E O Kasting G Leal R Neuroblastomycosis: report of three cases Arq Neuropsiquiatr 1968 26 01 73 79 10.1590/S0004-282x1968000100011

[19] Pereira W C Neves V J Rodrigues Y [Bifrontal decompressive craniotomy as the treatment for severe cerebral edema.] Arq Neuropsiquiatr 1977 35 02 99 111 10.1590/s0004-282x1977000200002 869742

[20] Pereira W C Tenuto R A Raphael A Sallum J [Brain localization of South American blastomycosis. Considerations apropos of 9 cases.] Arq Neuropsiquiatr 1965 23 02 113 126 10.1590/s0004-282x1965000200006 5851892

[21] Reimão R Lefévre A B Evaluation of flurazepam and placebo on sleep disorders in childhood Arq Neuropsiquiatr 1982 40 01 1 13 10.1590/s0004-282x1982000100001 7046699

[22] Scaff M Riva D Spina-França A [Schistosomal meningoradiculomyelopathy.] Arq Neuropsiquiatr 1971 29 02 227 233 10.1590/s0004-282x1971000200012 5159771

[23] Spina-França A Salum P NB Limongi J CP Berger A Losso E R [Myelopathies: diagnostic aspects.] Arq Neuropsiquiatr 1980 38 04 360 366 7469825

